# A Medical Miracle: Surviving a Perforating Gunshot Injury to the Neck

**DOI:** 10.7759/cureus.77713

**Published:** 2025-01-20

**Authors:** Rattan Singh, Jyoti Barwa, Apurba Patra, Sameer Peer, Ajay Kumar

**Affiliations:** 1 Department of Forensic Medicine and Toxicology, All India Institute of Medical Sciences, Bathinda, IND; 2 Department of Anatomy, All India Institute of Medical Sciences, Bathinda, IND; 3 Department of Diagnostic Radiology, All India Institute of Medical Sciences, Bathinda, IND

**Keywords:** bullet injury, gunshot, self-harm, temporary cavitation, wound ballistics

## Abstract

Gunshot wounds to the neck are considered among the most serious and life-threatening injuries due to the anatomical complexity of the neck and its vital structures. Surviving a perforating wound to the neck depends on various factors, including the location of the injury, the caliber of the weapon used, the type of bullet, the distance from which the patient was shot, prompt medical intervention, and the overall health of the individual. We report a case of miraculous survival following a perforating gunshot wound to the neck, in which no surgical intervention was required. Reconstruction of the bullet trajectory was performed using CT images and a virtual dissection table, Anatomage (Anatomage Inc., California, US), to facilitate the analysis of the factors that contributed to the patient’s survival.

## Introduction

Gunshot injuries are a significant cause of trauma worldwide. Penetrating or perforating gunshot wounds to the neck are considered one of the most dangerous and life-threatening injuries [1,2]. Moreover, the anatomical complexity of this region makes both the diagnosis and treatment of perforating wounds difficult. A penetrating injury to this area can lead to massive blood loss, airway obstruction, nerve damage, and spinal injury. The proximity of these structures means that a single bullet can cause multiple life-threatening injuries, often with fatal consequences [3].

There have been instances where people have survived gunshot wounds to the neck despite the vital structures packed into such small areas [4-6]. The ability to survive gunshot injury depends on the kind of tissue injured. For example, skeletal muscle, lungs, empty intestines, nerves, and blood vessels are more cohesive and elastic, allowing them to withstand the temporary cavitation caused by the bullet. In contrast, organs such as the liver, brain, and heart are less able to withstand the short-term cavitation produced by the bullet [7].

In gunshot wounds, internal injuries occur either from tissues being crushed by the bullet or its fragments, or from the pressure exerted on tissues due to temporary cavitation. Significant temporary cavitation is rare in gunshot wounds in urban settings, as most of these injuries result from less powerful handguns. The likelihood of surviving a gunshot wound to the neck also relies on variables, such as the injury's location, kind of bullet, weapon's caliber, patient's distance from the shooting, promptness of medical attention, and the person's general condition [4,8]. 

This paper aims to explore a case of a miraculous survival following a perforating gunshot wound to the neck, analyzing the factors that contributed to the patient’s survival with an emphasis on the need for complete psychiatric evaluation and counseling of such a patient.

## Case presentation

We report a case of a gunshot injury to the neck in an adult male who was brought to the emergency department of a tertiary care hospital within two hours of the incident by his father. According to the patient and his father, the patient was cleaning the muzzle of a pistol at home when ammunition was accidentally fired, resulting in injury to his neck.

On arrival at the hospital, the patient was conscious and oriented, with a GCS score of 15. The airway was patent, with spontaneous breathing and an oxygen saturation of 100%. There was no dyspnea, stridor, throat pain, or bleeding from the nose or throat.

**Figure 1:**
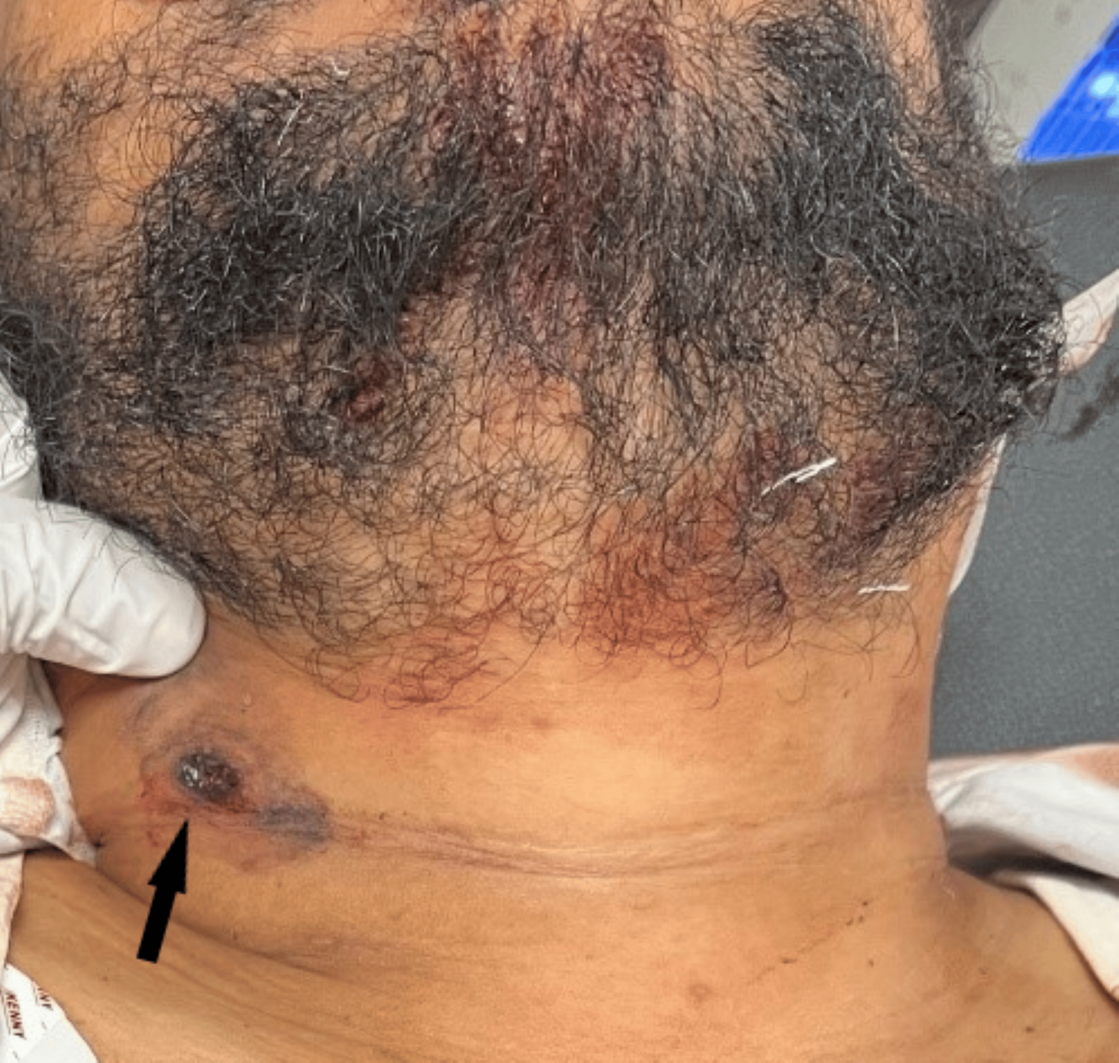
Entry wound caused by a rifled firearm

**Figure 2:**
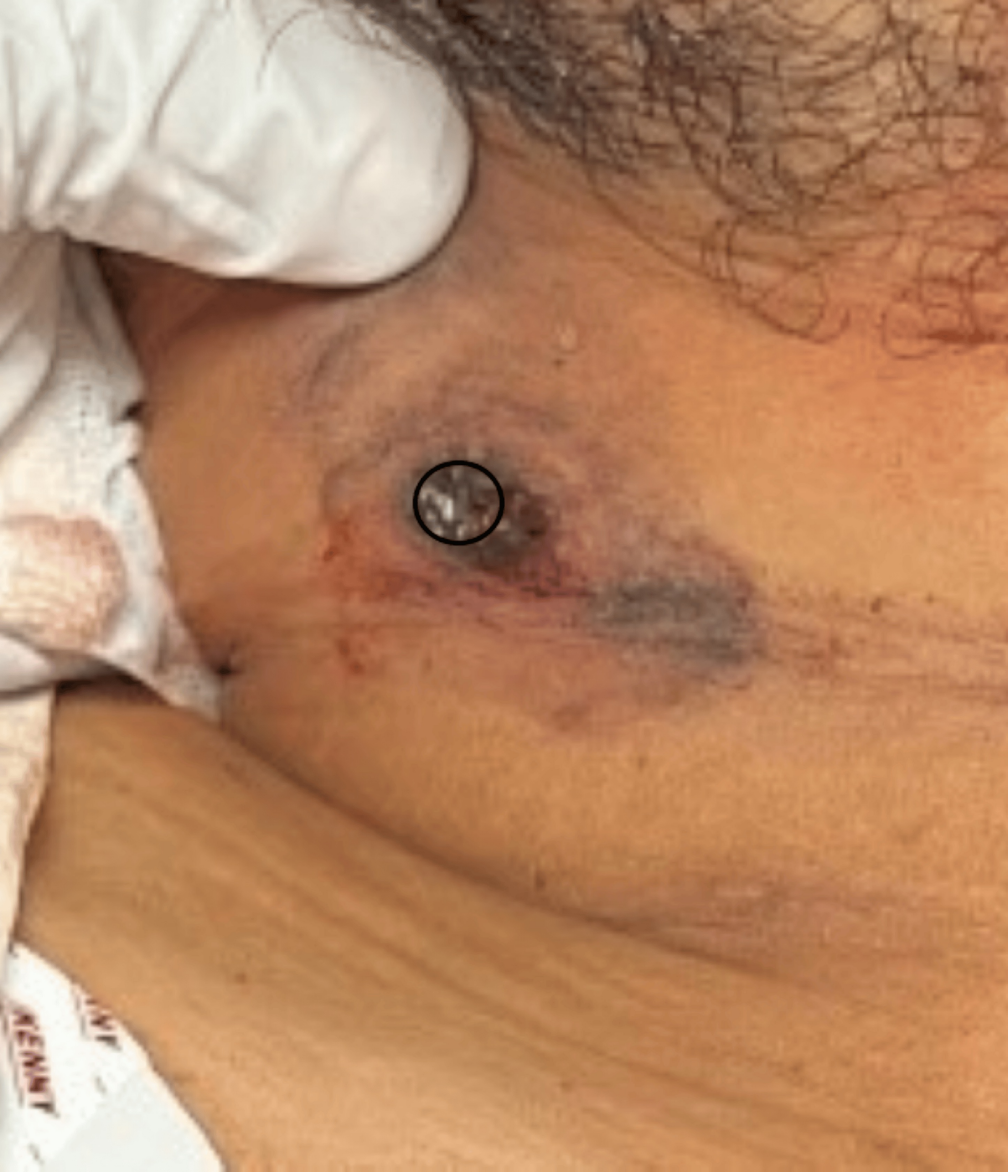
Magnified image of firearm entry wound showing lead ring

**Figure 3:**
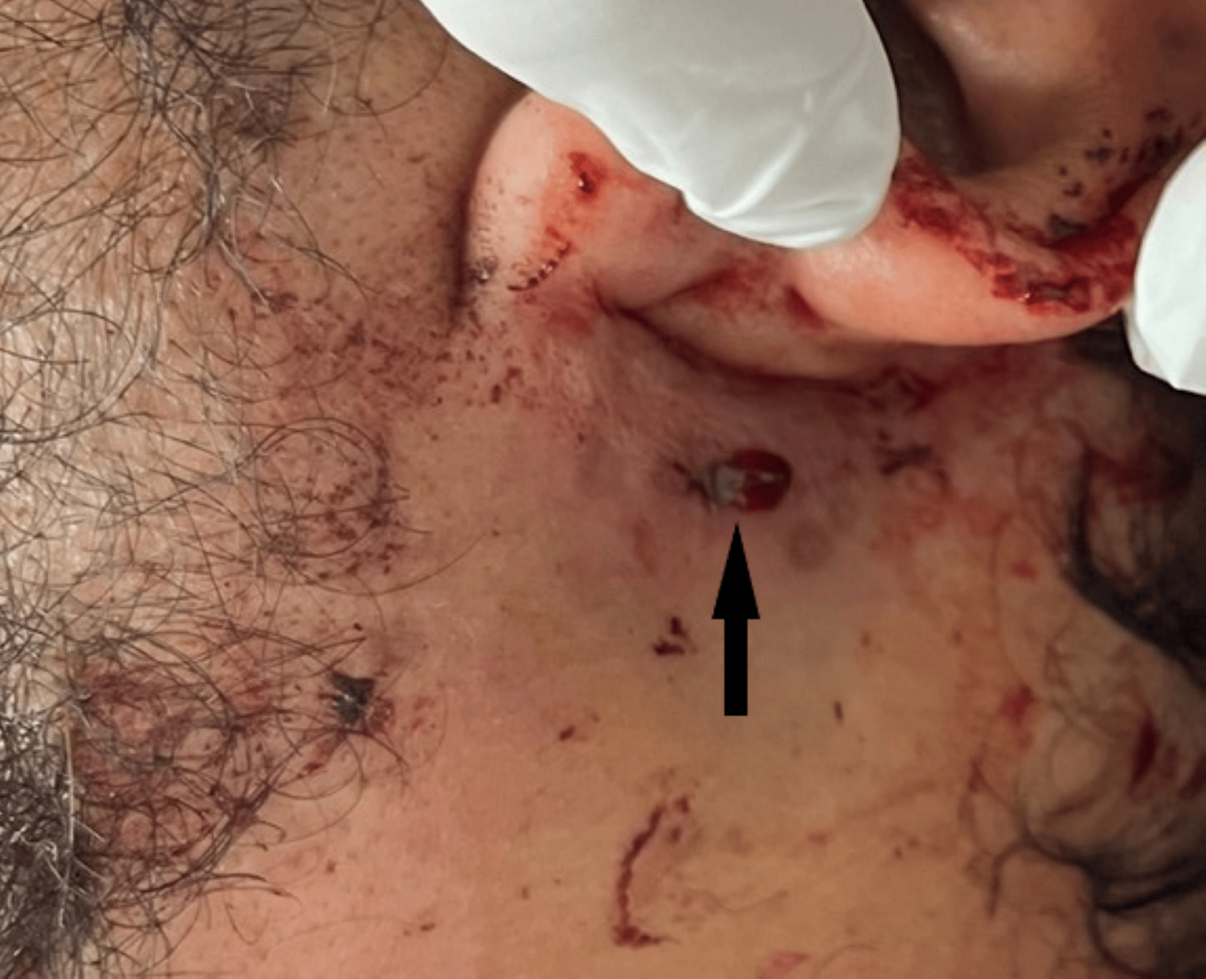
Exit wound of the rifled firearm

On local examination, a circular firearm entry wound with inverted margins, measuring 1.1 cm x 1 cm, was observed on the outer aspect of the right side of the neck, situated 3.5 cm below the jawline (Figure 1). An abrasion collar was present around the outer aspect of the entry wound, with blackening noted circumferentially. Additionally, a lead ring was incompletely visualized (Figure 2). The exit wound, with everted margins and measuring 0.6 cm x 0.5 cm, was present on the left side of the neck, situated 1 cm below the angle of the jaw (Figure 3). The pinna, preauricular, and postauricular areas showed no abnormalities. There was no mastoid tenderness, and the tragal sign was negative, with a clear external auditory canal and intact tympanic membranes on both sides. The motor function of the facial nerve was intact on both sides. No abnormalities were detected in the oral cavity. On laryngeal endoscopy, a minor laceration was noted on the posterior pharyngeal wall.

Contrast-enhanced computed tomography (CECT) head, neck, and thorax were performed immediately, which revealed no foreign body mass anywhere in the scan. Air was noted in anterior and posterior cervical spaces, retropharyngeal, bilateral parapharyngeal, right submandibular, and right buccal space. A focal ulcerated area was noted in the posterior pharyngeal wall. However, there was no evidence of perforation of any vital structures of the neck. Brain parenchyma was also within normal limits.

At the entry point, the bullet penetrated the right sternocleidomastoid (SCM) muscle. It then traveled in a superolateral direction, passing behind the greater cornu of the hyoid bone. In the mid-neck region, the bullet traversed the space located posterior to the pharyngeal wall and anterior to the anterior longitudinal ligament at the C3 vertebral level, known as the space of Gillette. Subsequently, it traveled superolateral behind the posterior edge of the left mandibular ramus. Finally, the bullet passed through the left parotid gland without causing any damage to the facial nerve and exited the body piercing the subcutaneous tissue and skin.

After a complete assessment, no surgical intervention was carried out as there was no damage to any vital neck structures; monitoring of the patient was done along with stabilization of vitals and oxygen supplementations, surgical dressing of the external wounds was also done, and medication in the form of intravenous fluids, antibiotics, antipyretics, steroids, and painkiller was administered. However, on psychiatric evaluation, it was discovered that the patient was experiencing property-related conflicts with his wife and in-laws, which led to self-harm and suicidal thoughts; the firing incident was actually an impulsive suicidal attempt and not accidental in nature. There was also a history of an increase in the frequency and quantity of alcohol consumption over the past three months. After four days of medical management, the patient was shifted to a psychiatric ward with strict supervision under the care of his family members and advised the adoption of high-risk measures by hospital staff; he was subjected to counseling sessions on a daily basis along with antipsychotics, anticonvulsants, antidepressants, and other mood stabilizers.

The patient was discharged after two weeks of observation and advised to follow up in the psychiatry and ENT departments on an outpatient basis. This case presents an extraordinary example of survival following a perforating gunshot wound to the neck, not warranting much medical care or rehabilitation.

## Discussion

Categorization and management of neck wounds can be done based on the division of the neck into three anatomic zones [9]. Zone I reaches the clavicles and thoracic outlet from the base of the cricoid cartilage. The trachea, esophagus, upper mediastinum, lung apices, thoracic duct, and major vessels are all located within this zone. Zone II encompasses the region between the mandibular angle and the cricoid cartilage. It includes the pharynx, larynx, esophagus, trachea, jugular veins, and carotid and vertebral arteries. Zone III comprises the distal extracranial carotid and vertebral arteries, as well as portions of the jugular veins, and extends from the jaw's angle to the skull's base.

Penetrating neck injuries (PNIs) or perforating neck injuries can occur across zone boundaries depending on the projectile or instrument and its angle of penetration, therefore trauma to the neck is not always restricted to a single zone [4]. In this case of perforating neck injury, the bullet entered Zone I on the right side, traversed through Zone II, and exited from Zone III on the left side of the neck.

The mortality rate associated with penetrating neck trauma can reach 11%. In two-thirds of all cases, injuries to key structures could be fatal [5]. Although Zone II accounts for the majority of injuries, Zone I has the greatest mortality rate [10]. Exsanguination is the most common cause of mortality in these circumstances because vascular injuries, of which carotid and internal jugular injuries are the most common, account for up to 25% of structural injuries. Since the vertebral artery is in a protected anatomical position, it is less frequently affected. The respiratory tract is implicated in 10% of cases, necessitating effective airway management to prevent respiratory complications [9]. In this case, since none of the vital structures in either of the zones of the neck was damaged, the GCS score of the patient remained 15/15 throughout the hospital stay, and no acute medical emergency was warranted.

Studies recommend a novel management protocol with a "no zone" approach for stable patients, comprising early radiological evaluation utilizing CT-angiography, for effective and safe management, thereby minimizing negative neck exploration rates [11,12]. In the absence of active bleeding from the entry or exit wound in our case, CT-angiography was not performed.

**Figure 4:**
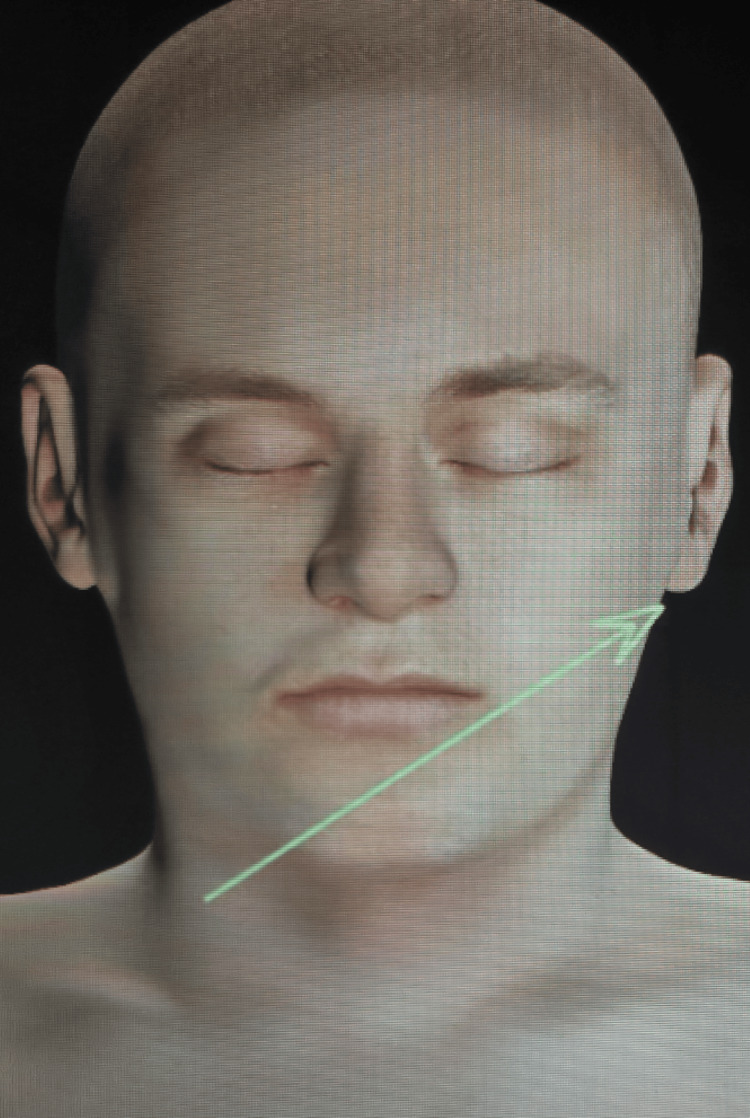
Depiction of the wound tract on the body surface 3D images were created from DICOM images of CECT scans of the head, neck, and thorax using the Anatomage table to determine the bullet's direction of the bullet. CECT: contrast-enhanced computed tomography

**Figure 5:**
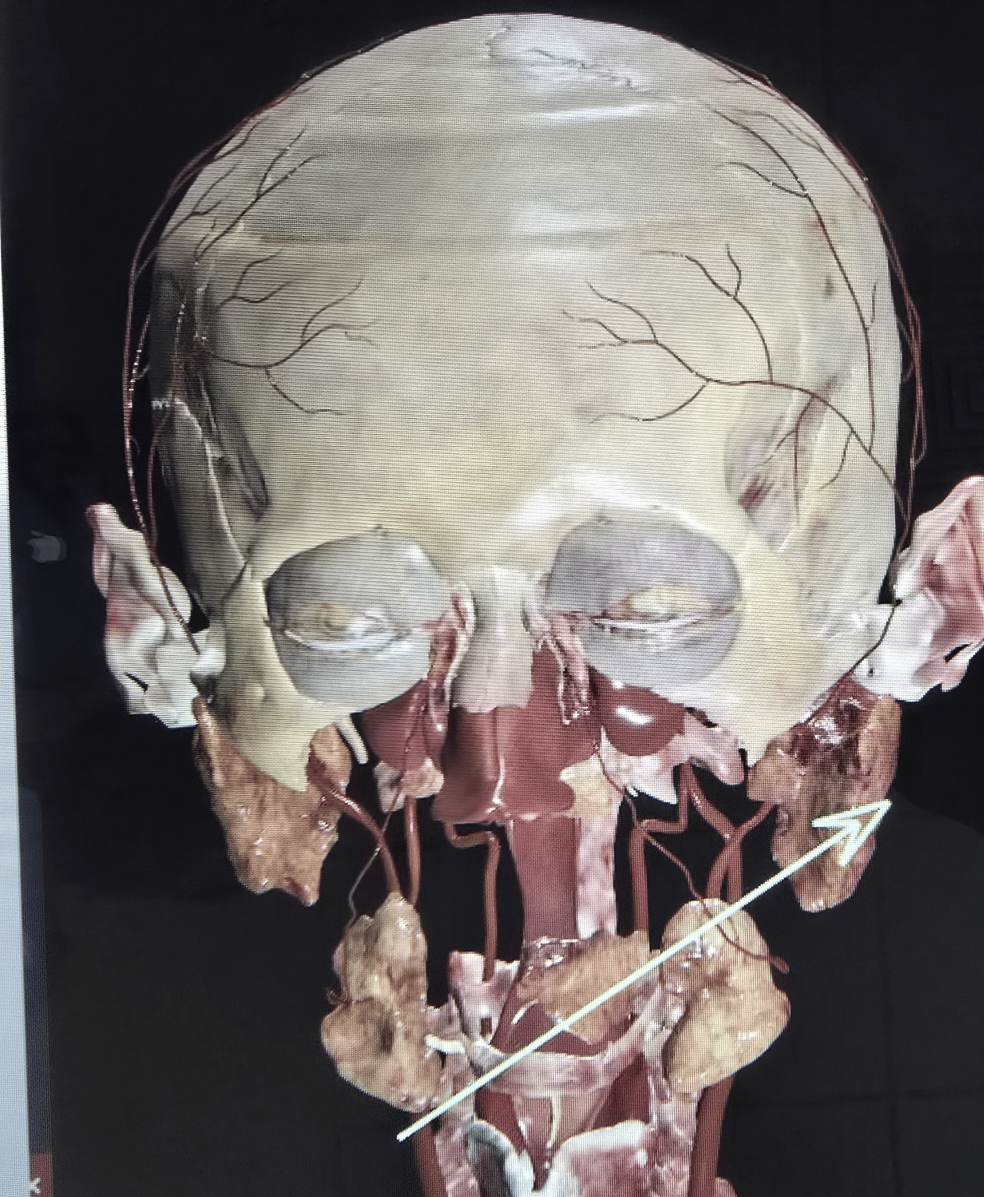
Site of entry and exit with structures traversed

**Figure 6:**
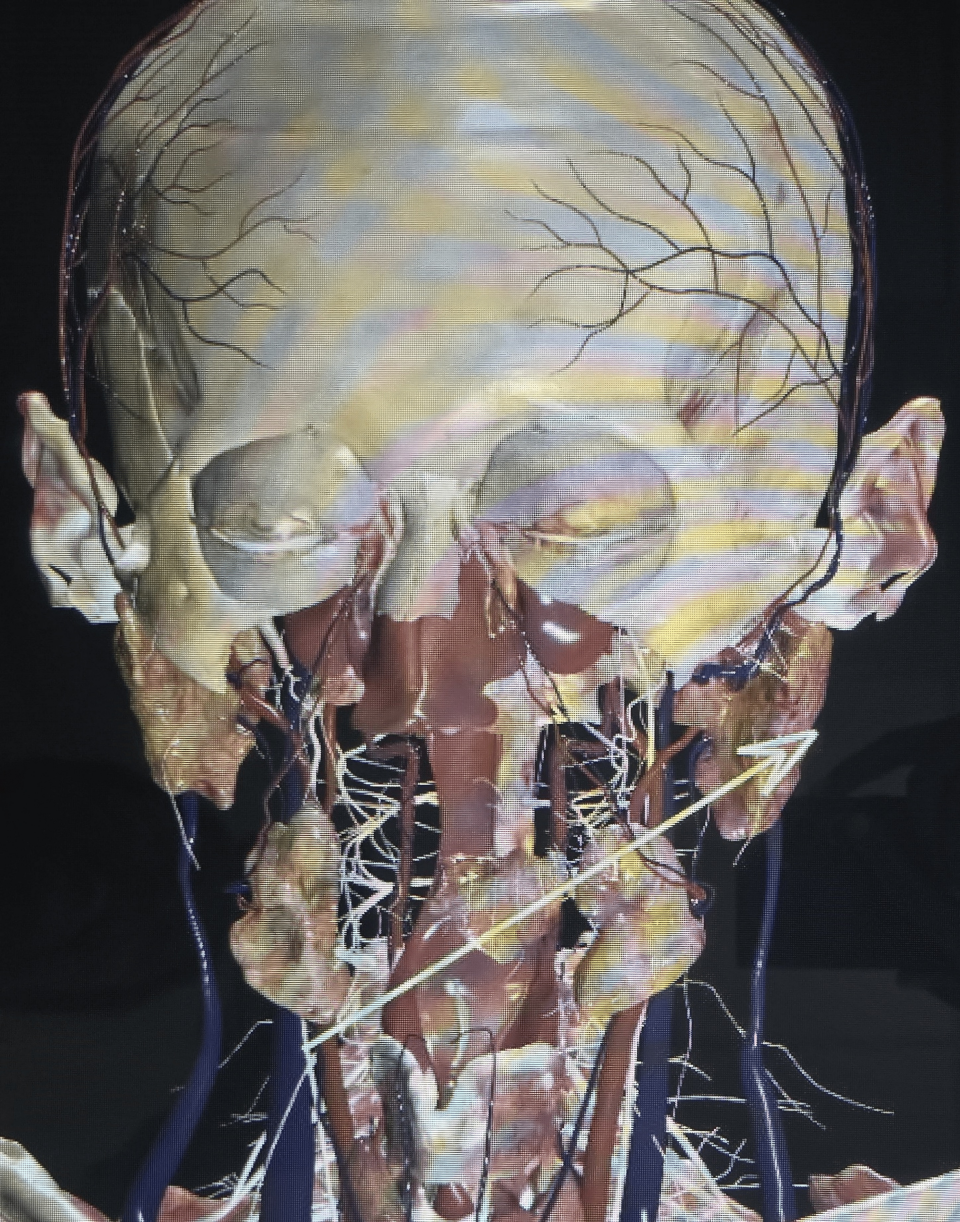
After the removal of the mandible, maxilla, and other bony structures of the lower half of the face

**Figure 7:**
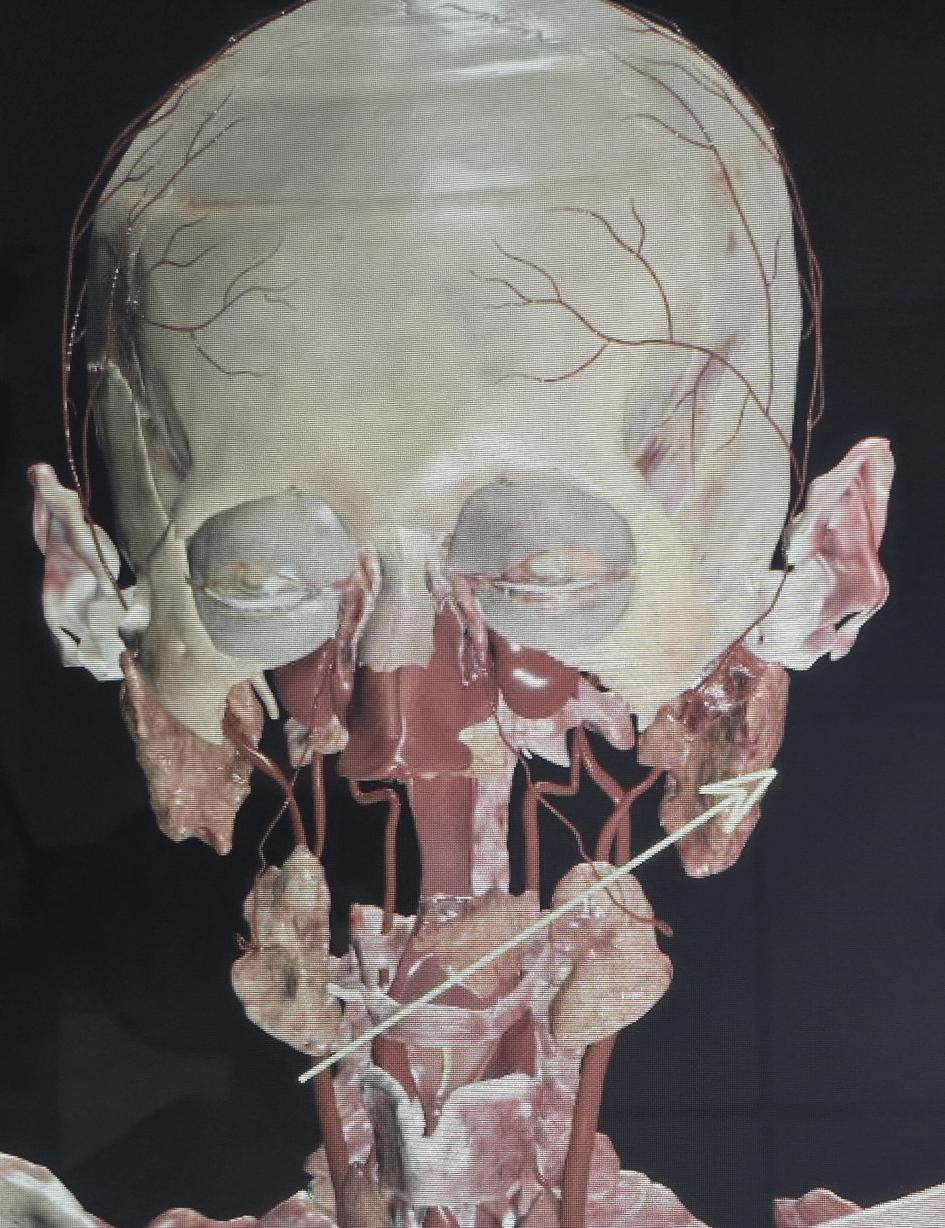
After removal of the facial nerves and veins

The authors have considered the following factors that contributed to the patient's miraculous survival from a firearm injury to the neck. Type of firearm: the firearm was a centrefire handgun, specifically a 9 mm licensed pistol, discharged as a near-contact gunshot wound. A centrefire rifle or handgun is typically required to fire a bullet capable of causing significant temporary cavitation, resulting in tissue destruction [8]. However, in this case, despite the similar firearm used, the temporary cavitation did not cause massive damage to the surrounding structures because the site was supported posteriorly by the vertebral column and prevertebral fascia, and anteriorly by the flexible part of the pharynx. Position of the victim: the patient was leaning forward with the pistol in his right hand, potentially increasing the retropharyngeal space (RPS), which spans from the skull base to the upper mediastinum, while the prevertebral space (PVS) extends from the skull base to the coccyx. The danger space, located posterior to the RPS, is also known as the prevertebral, anterior visceral, or vascular visceral space, and is comprised of loose areolar tissue defined by the alar fascia anteriorly and the prevertebral fascia posteriorly [10,13-15]. The bullet traversed through this space, sparing all vital structures. Bullet trajectory: the trajectory of the bullet played a crucial role in sparing vital structures, such as the carotid artery, jugular vein, and spinal cord. The trajectory was reconstructed using CT films and the Anatomage table and software (Anatomage Inc., California, US) (Figure 4, 5, 6, 7). Wound ballistics: contrary to the smaller entry wounds typically observed, the entry wound in this case was larger than the exit wound. This can occur due to the dynamics of a bullet’s behavior as it travels through the body, influenced by factors such as the bullet's caliber, velocity, shape, and interaction with body tissues [8]. The larger entry wound could be attributed to the handgun used, which possibly had limited wounding potential, or to the bullet losing significant energy during its passage, leaving insufficient energy at the exit to cause substantial damage, yet still enabling it to exit the body.

## Conclusions

This report demonstrates remarkable survival from perforating gunshot wounds to the neck with no significant injury to vital structures in an adult male. However, rapid assessment, advanced imaging, and appropriate medical management have been shown to play an important role in stabilizing the patient and preventing complications. Additionally, psychiatric evaluation revealed underlying mental health issues, including suicidal ideation, necessitating a multidisciplinary approach by psychiatry for long-term care. The case highlights the importance of addressing psychosocial factors contributing to such injuries and emphasizes that both medical and psychological support must be provided during recovery and thereafter to ensure optimal recovery and prevent recurrence.

## References

[1] Nowicki JL, Stew B, Ooi E (2018). Penetrating neck injuries: a guide to evaluation and management. Ann R Coll Surg Engl.

[2] Brywczynski JJ, Barrett TW, Lyon JA, Cotton BA (2008). Management of penetrating neck injury in the emergency department: a structured literature review. Emerg Med J.

[3] Kazi M, Junaid M, Khan MJ, Ali NS, Masoom A (2013). Utility of clinical examination and CT scan in assessment of penetrating neck trauma. J Coll Physicians Surg Pak.

[4] Godhi S, Mittal GS, Kukreja P (2011). Gunshot injury in the neck with an atypical bullet trajectory. J Maxillofac Oral Surg.

[5] Pippal SK, Soni S, Asif SK, Bhadoria S (2009). An extraordinary case of ricochet gunshot injury in the head & neck region with an atypical bullet trajectory: a case report. World Articles Ear Nose Throat.

[6] Kaufman EJ, Wiebe DJ, Xiong RA, Morrison CN, Seamon MJ, Delgado MK (2021). Epidemiologic trends in fatal and nonfatal firearm injuries in the US, 2009-2017. JAMA Intern Med.

[7] Malcolm JD (2006). Terminal Ballistics: A Text and Atlas of Gunshot Wounds. https://doi.org/10.1201/9781420037463.

[8] Baum GR, Baum JT, Hayward D, MacKay BJ (2022). Gunshot wounds: ballistics, pathology, and treatment recommendations, with a focus on retained bullets. Orthop Res Rev.

[9] Moeng S, Boffard K (2002). Penetrating neck injuries. Scand J Surg.

[10] Phan T, Lay J, Scali F (2022). The alar fascia and danger space: a modern review. Cureus.

[11] Motamedi MH (2003). Primary management of maxillofacial hard and soft tissue gunshot and shrapnel injuries. J Oral Maxillofac Surg.

[12] Chandrananth ML, Zhang A, Voutier CR, Skandarajah A, Thomson BN, Shakerian R, Read DJ (2021). 'No zone' approach to the management of stable penetrating neck injuries: a systematic review. ANZ J Surg.

[13] Grodinsky M, Holyoke E (1938). The fasciae and fascial spaces of the head, neck and adjacent regions. Am J Anat.

[14] Casberg MA (1950). The clinical significance of the cervical fascial planes. Surg Clin North Am.

[15] Chong VF, Fan YF (2000). Radiology of the retropharyngeal space. Clin Radiol.

